# Advancing cybersecurity and privacy with artificial intelligence: current trends and future research directions

**DOI:** 10.3389/fdata.2024.1497535

**Published:** 2024-12-05

**Authors:** Krishnashree Achuthan, Sasangan Ramanathan, Sethuraman Srinivas, Raghu Raman

**Affiliations:** ^1^Center for Cybersecurity Systems and Networks, Amrita Vishwa Vidyapeetham, Amritapuri, Kollam, Kerala, India; ^2^School of Engineering, Amrita Vishwa Vidyapeetham, Coimbatore, India; ^3^School of Engineering and Computer Science, University of Pacific, Stockton, CA, United States; ^4^School of Business, Amrita Vishwa Vidyapeetham, Amritapuri, Kollam, Kerala, India

**Keywords:** artificial intelligence, cybersecurity, privacy, topic modeling, ethics, quantum, explainable AI, cryptography

## Abstract

**Introduction:**

The rapid escalation of cyber threats necessitates innovative strategies to enhance cybersecurity and privacy measures. Artificial Intelligence (AI) has emerged as a promising tool poised to enhance the effectiveness of cybersecurity strategies by offering advanced capabilities for intrusion detection, malware classification, and privacy preservation. However, this work addresses the significant lack of a comprehensive synthesis of AI's use in cybersecurity and privacy across the vast literature, aiming to identify existing gaps and guide further progress.

**Methods:**

This study employs the Preferred Reporting Items for Systematic Reviews and Meta-Analyses (PRISMA) framework for a comprehensive literature review, analyzing over 9,350 publications from 2004 to 2023. Utilizing BERTopic modeling, 14 key themes in AI-driven cybersecurity were identified. Topics were clustered and validated through a combination of algorithmic and expert-driven evaluations, focusing on semantic relationships and coherence scores.

**Results:**

AI applications in cybersecurity are concentrated around intrusion detection, malware classification, federated learning in privacy, IoT security, UAV systems and DDoS mitigation. Emerging fields such as adversarial machine learning, blockchain and deep learning are gaining traction. Analysis reveals that AI's adaptability and scalability are critical for addressing evolving threats. Global trends indicate significant contributions from the US, India, UK, and China, highlighting geographical diversity in research priorities.

**Discussion:**

While AI enhances cybersecurity efficacy, challenges such as computational resource demands, adversarial vulnerabilities, and ethical concerns persist. More research in trustworthy AI, standardizing AI-driven methods, legislations for robust privacy protection amongst others is emphasized. The study also highlights key current and future areas of focus, including quantum machine learning, explainable AI, integrating humanized AI and deepfakes.

## 1 Introduction and related work

In the fast-paced digital era, artificial intelligence (AI) has greatly transformed various sectors (Vinuesa et al., 2020), and its role in cybersecurity is becoming increasingly important. The traditional paradigms of cybersecurity are constantly being tested as cyber threats become more sophisticated, demanding advanced solutions that can respond to and prevent attacks (Salem et al., 2024). This research paper explores how AI is integrated into cybersecurity and privacy strategies—an encouraging method for defending digital fortresses. AI's capabilities, including predictive analytics, machine learning, and autonomous decision-making, allow it to go beyond merely detecting threats but also have a proactive approach against threat actors in cyberspace (Zhang et al., 2022). By using advanced ML algorithms, patterns and anomalies can be identified from large datasets to detect potential future threats before they become full-blown attacks (Sarker, 2023; Kaur et al., 2023). It enables systems to grow and learn from their experiences without explicit programming (Macas et al., 2022; Gugueoth et al., 2023). In this scenario, where reactive actions are often not timely enough to protect against significant damage, this predictive ability of AI becomes essential. Furthermore, AI systems can change and improve themselves when there are new risks (Gambín et al., 2024), making them more effective than static rule-based systems. Nonetheless, the use of artificial intelligence in cybersecurity has come with its challenges. There are concerns about issues such as lack of transparency in AI decision-making processes (Stevens, 2020), sophisticated hackers who may manipulate or bypass these systems, according to Guembe et al. (2022), and ethical considerations regarding autonomy in cyber defense, according to Landini (2020). Recent years have seen a growing interest in generative artificial intelligence and the ability to increase the threat landscape considerably (Raman et al., 2024d). For AI-driven cybersecurity measures to be accepted and effective, their dependability, transparency, and accountability must be assured.

The current trend in digital threats portrays a grim reality. Researchers have demonstrated the ongoing development of adversaries with ties to nation-states and criminal organizations (Chithaluru et al., 2023). With the increasing complexity of cyberattacks, new and invasive techniques (Loggen et al., 2024) are being developed to identify even the most impeccable targets. The significance of robust cybersecurity measures cannot be overstated (Mishra et al., 2022), as they serve as anchors for safeguarding individuals (Achuthan et al., 2023), information systems, and networks against the backdrop of an increasingly hostile digital landscape. The nature of cyber threats is continually evolving, with attackers leveraging sophisticated techniques such as artificial intelligence (AI) and machine learning to conduct more effective and stealthy operations (Alshahrani et al., 2022). These advanced persistent threats (APTs) represent a significant challenge because they are designed to evade traditional security measures and persist within networks for extended periods (Zhang et al., 2021; Merzouk et al., 2022). To address these multifaceted threats, cybersecurity strategies must evolve to incorporate not only advanced technologies but also a deeper understanding of the human elements involved in cybersecurity. The rise of ransomware (Oz et al., 2022; Alkhalil et al., 2021), and state-sponsored cyber espionage further complicates the security landscape, making the role of AI in cybersecurity not only advantageous but also essential.

Numerous studies have been conducted to explore the dynamic aspects of cybersecurity research, with particular emphasis on the role of artificial intelligence (AI) in this field. A number of reviews (Table 1) have shown the extent and thematic focus of cyber security research, notably pointing to increased adoption of AI as a means to strengthen cyber threat intelligence. Zhang et al. (2022) review 131 papers, primarily highlighting established AI techniques like SVM, CNN, and ANN. While their conceptual AI-driven cybersecurity framework introduces a promising human-AI hybrid approach, the study overlooks emerging AI models better suited for scalability and adaptability. The framework, however, lacks actionable guidance on real-world implementation challenges, such as resource constraints and integrating human expertise into automated systems. They also note gaps in AI's adaptability to evolving threats, underscoring the current need for systems that dynamically adjust to new attack landscapes. Similarly, Wiafe et al. (2020) and Abdullahi et al. (2022) map AI applications in cybersecurity, identifying intrusion detection and prevention as the most studied areas and surveying widely used techniques. Macas et al. (2022) examine deep learning (DL) applications in cybersecurity across 80 studies, emphasizing DL's strengths in handling complex data for intrusion detection, malware analysis, and encrypted traffic analysis. Guembe et al. (2022) discuss AI's dual role in cybersecurity, noting both its defensive potential and its use in enhancing cyberattacks, such as spear-phishing and automated evasion. Their study, however, focuses solely on DL, leaving other AI methods unaddressed. Salem et al. (2024) provide a broad review of ML, DL, and metaheuristic algorithms across recent studies, stressing AI's ability to automate detection and process large datasets. Despite the comprehensive analysis, practical insights into real-world scalability are limited. Similarly, Eke and Onyejegbu (2023) and Li et al. (2020) use bibliometric methods to quantify trends in AI cybersecurity research, analyzing 340 and 100 publications, respectively. These studies highlight AI's strengths and limitations but lack in-depth exploration of application challenges such as privacy concerns, UAV integration, and resource constraints. Regarding the role of artificial intelligence in specific applications and sectors, such as those by Campedelli (2021) and Bolbot et al. (2022), they shed light on the application of AI in crime research and emerging areas such as the maritime industry. These studies reveal the specialized nature of cybersecurity concerns across different fields, yet they also indicate potential gaps in the research, such as the under representation of ethical considerations in AI and crime research and the need for a more comprehensive exploration of the maritime industry's cybersecurity evolution.

**Table 1:** Summary of related studies.

**References**	**Years of study**	**Articles**	**Scope of study**	**Application domains covered**
Wiafe et al. (2020)	2008–2018	131	AI Methods in Cybersecurity	Intrusion detection, encryption, and certification, imaging and captcha, Phishing and malware, Traffic classification, DoS
Zhang et al. (2022)	2016–2020	54	User access authentication, Network situation awareness	User access authentication, Network situation awareness, Dangerous behavior monitoring, and Abnormal traffic identification and their accuracy
Abdullahi et al. (2022)	2016–2021	80	Deep learning (DL) and Machine learning (ML) techniques are used in IoT security along with Model performance.	IoT Domain (AI-based methods classified as Probe, U2R, R2L, DoS)
Macas et al. (2022)	2016–2021	65	DL for Cybersecurity	Malware Detection and Analysis, Fraud Detection, Spam Filtering, Cyber-Physical System (CPS) Attack Detection, Botnet Detection and DGAs, Encrypted Traffic Analysis, Network Intrusion Detection, and Authentication Solutions
Guembe et al. (2022)	Not Specified	46	AI-Based Attacks (Reconnaissance, Access and penetration, Delivery, Exploitation, Command and control, Action on objectives)	AI-Driven Attacks in the Access and Penetration stage, delivery stage, exploitation stage, command and control stage of the Cybersecurity Kill Chain
Eke and Onyejegbu (2023)	2007–2023	340	AI and Machine Learning Techniques	Network intrusion, Phishing detection
Li et al. (2020)	1999–2018	1,000	Medical image processing, SDN, DL, IDS, Blockchain, Organization threat assessment, SDN, Cryptographic advancements, Wireless communication	General Information on Research Hotspots
Salem et al. (2024)	2020–2024	68	Studies utilizing ML, DL, or Metaheuristic (MH) algorithms	Evaluation of open access cyber-security datasets

Overall, while these papers collectively provide a rich overview of current trends and developments in cybersecurity and AI, they also highlight the need for broader, more in-depth analyses that encompass a wider range of AI methodologies through the analysis of a larger cohort of articles and the practical impacts of research on advancing cybersecurity solutions. As cyber threats grow more sophisticated, conventional computational power may not suffice for handling the high-dimensional data and complex threat landscapes associated with modern attacks. Quantum computing, with its significantly enhanced processing capabilities, presents a promising frontier to enhance AI's potential in cybersecurity. By leveraging quantum algorithms (Khurana, 2024), more highly efficient encryption, rapid threat pattern analysis, and predictive capabilities that surpass classical AI methods are possible.

To address the some of the challenges at the intersection of AI and cybersecurity and privacy, a broader examination of existing research is crucial. This will highlight key areas of progress, summarize challenges, and shape a comprehensive roadmap for future research and practice. Such an overview is essential for understanding the current landscape, anticipating future trends, and preparing for emerging threats. By integrating insights from various cybersecurity and AI subfields, researchers and practitioners can better grasp the synergies and challenges within this critical intersection. Accordingly, we propose the following research questions.

1. RQ1: What are the main areas where AI is applied in cybersecurity and privacy, how are these applications distributed across specific topics and evolved over time?
2. RQ2: What are the thematic relationships and similarities between key AI-driven cybersecurity and privacy topics? What is the global distribution of research contributions?
3. RQ3: What are the major researched topics over the two decades? What are their strengths and limitations?
4. RQ4: What are the emerging challenges and underexplored areas that require advanced methodologies and interdisciplinary approaches?

Our study distinguishes itself from previous work by offering the following unique contributions. First, the study exploits a broad dataset of almost 10,000 publications between 2004 and 2023 that provides an overview of the cybersecurity and privacy domain, with a particular emphasis on AI. Second, utilizing the BERTopic method, the research classifies numerous topics related to cybersecurity into 14 categories to provide a well-structured and informed exploration of the respective field. Third, AI advancements in this area are approached from different perspectives by this article to reveal various unique aspects of its application within security systems that help to expose links and specializations inside it. Fourthly, this study identified four key patterns and contributions through a detailed examination of scientific articles covering issues related to cybersecurity and countrywide distribution across the globe. Finally, future areas of critical research interest are identified and discussed in the conclusion, which offers insights that can guide research on how some fields develop stronger security measures over time. Thus this study addresses both current AI applications and prospective quantum advancements, toward strengthening cybersecurity resilience.

## 2 Methods

### 2.1 PRISMA framework

**Figure 1:**
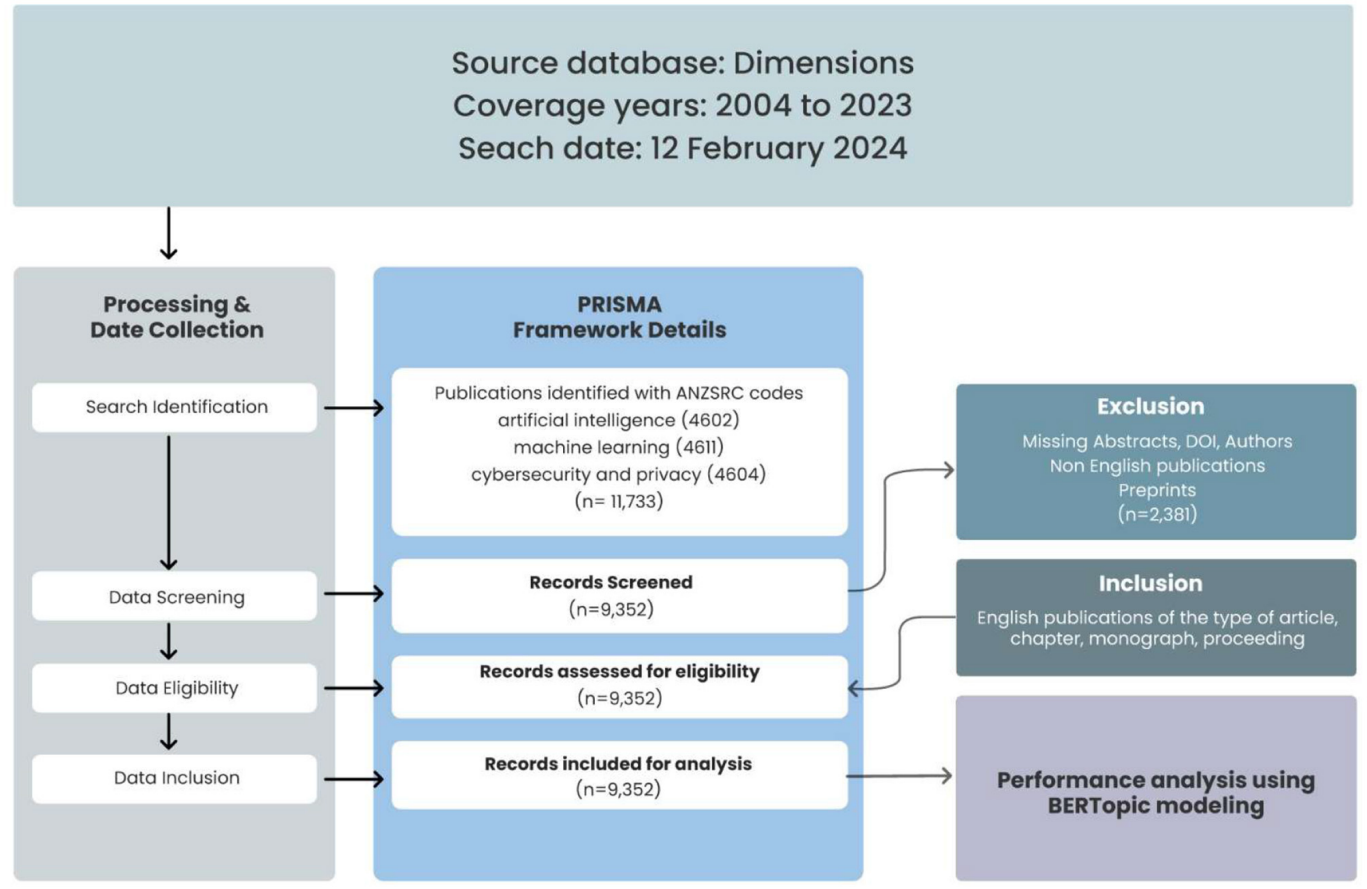
Research framework based on PRISMA.

The analysis of this study was guided by the Preferred Reporting Items for Systematic Reviews and Meta-Analyses (PRISMA) framework as stipulated by Page et al. (2021), as seen in Figure 1. The PRISMA framework consists of a structured five-step process for identifying research questions; constructing comprehensive search strategies that include databases, search terms, and inclusion/exclusion criteria; and conducting literature searches and screening the literature on titles, abstracts and full texts before conducting the analysis. This organized approach allows for the selection and evaluation of studies to be included in systematic reviews or meta-analyses; hence, this approach can be used across disciplines such as economics (Wang and Groene, 2020), e-learning (Behl et al., 2022), and sustainable development goals (Raman et al., 2023, 2024a,c). The identification phase began with a literature search in the Dimensions database on February 12, 2024, between 2004 and 2023. This database was chosen because it has a wide coverage of journals, which is far greater than that of other databases, such as Scopus, whose journal coverage is < 48.1%, or Web of Science, whose journal coverage is far less than approximately 82.2% compared to that of the Dimensions database (Raman et al., 2024b). The Dimensions database utilizes the Australian and New Zealand Standard Research Classification (ANZSRC) system, which is organized into 22 broad Fields of Research (FoR) divisions, allowing for detailed classification of research activities. A single publication can be categorized under multiple Fields of Research within the ANZSRC system. Using the ANZSRC framework (Raman, 2023), a total of 11,733 publications on artificial intelligence (ANZSRC 4602), machine learning (ANZSRC 4611), and cybersecurity and privacy (ANZSRC 4604) were identified. At the screening level, only English publications of the type of article, chapter, monograph, and proceeding were retained for a total of 9,352 publications, which were included for final analysis. Publications with missing abstracts, author details, DOIs, and preprints were excluded.

### 2.2 BERTopic modeling

Topic modeling offers various techniques, such as Non-negative Matrix Factorization (NMF), Latent Dirichlet Allocation (LDA), Probabilistic Latent Semantic Analysis (PLSA), and To2Vec. While effective, these methods often fail to capture semantic relationships between words and struggle with short-text data (Egger and Yu, 2022). Traditional topic modeling approaches, like those based on the Bag-of-Words (BoW) model, prioritize term frequency but lack deeper semantic insights. In contrast, BERTopic leverages embeddings (Grootendorst, 2022) to represent documents in a lower-dimensional space, enhancing the contextual understanding of the content (Alamsyah and Girawan, 2023; Raman et al., 2024c). This method is built on BERT, a bidirectional language model developed by Google that processes language in a context-aware manner (Devlin et al., 2019).

Modeling begins with embedding vectorization, which involves converting input text into numerical embeddings. This is followed by dimensionality reduction, where these embeddings are streamlined using Unified Manifold Approximation and Projection (UMAP). UMAP clusters similar data points, creating more distinct topic groups (Yi et al., 2025). After reducing the data, Hierarchical Density-Based Spatial Clustering of Applications with Noise (HDBSCAN) is applied to identify clusters by grouping densely packed data points while ignoring outliers. To interpret these clusters, the model employs class-based Term Frequency-Inverse Document Frequency (c-TF-IDF), which highlights significant words or phrases for each group, allowing us to recognize and rank topics within the dataset (Oh et al., 2024). For this analysis, we used “all-MiniLM-L6-v2” as the default text representation model, optimized for clustering and semantic search in English (Kim et al., 2024). This enhanced version of TF-IDF identifies key terms by weighing their frequency within a document against their rarity across the corpus, thus assigning topics to documents based on these terms with associated probabilities indicating each document's topic relevance (Khodeir and Elghannam, 2024).

The UMAP algorithm was configured with default parameters, including setting “calculate probabilities” to “True” to estimate document-topic associations and the language specified as English. The topic count was determined through both quantitative and qualitative evaluations, focusing on intertopic distance and coherence scores. We experimented with topic numbers ranging from 4 to 20, with a minimum distance parameter of 0.05, to ensure adequate separation within the reduced-dimensional space. The “cosine” metric was used to assess the angular similarity between vectors, and a random state of 100 was chosen to maintain reproducibility. Additionally, the nearest neighbor parameter (n_neighbors) was set to 15, allowing the model to emphasize the local neighborhood of data points, thus preserving finer details while also capturing larger patterns (McInnes et al., 2018). This configuration yielded 14 main topics, with 20 representative publications identified for each.

Although machine learning techniques are effective for data clustering, the risk of misclassification persists (Lyutov et al., 2024). To enhance the accuracy and interpretability of the outcomes, a manual review was conducted on both the topics and their representative publications. This qualitative assessment involved three experts who evaluated the cohesiveness and relevance of each topic, as supported by recent studies applying BERTopic (Douglas et al., 2024; Wang et al., 2023). The experts reviewed topic keywords and representative publications by examining abstracts, titles, and, when necessary, full papers. They also considered probability values and citation counts to select the top three representative articles for each topic. This review ensured that the unsupervised topic modeling results were both precise and practical, aiding in the identification of major themes in the dataset. Following this process, the original 14 topics were retained, and a deeper analysis was conducted on the top three representative publications (based on citations) within each topic.

## 3 Results and discussion

### 3.1 Distribution of topics (RQ1)

**Figure 2:**
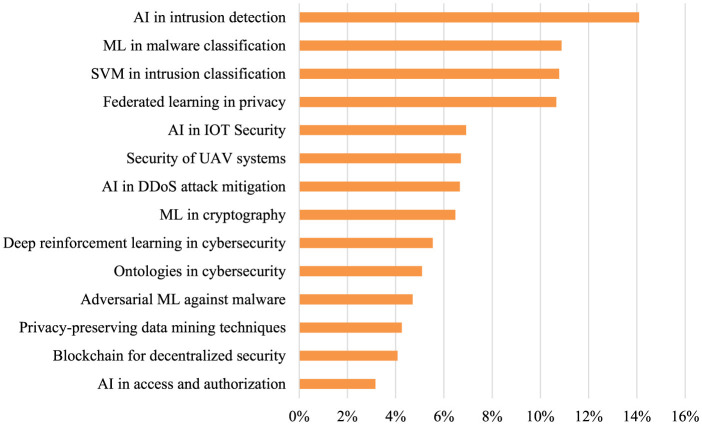
Major topics extracted and their proportions.

Figure 2 presents the distribution of key topics within the analyzed publications, offering a clear perspective on the areas where AI contributes to cybersecurity and privacy. Intrusion detection using AI leads as the most prominent focus area, comprising approximately 13% of the total content analyzed. This suggests a widespread emphasis on using AI to identify and respond to unauthorized system access. Malware classification, specifically utilizing machine learning (ML) techniques, follows closely at around 10%, reflecting growing concerns over malicious software like ransomware and viruses in the cybersecurity domain. In terms of privacy, federated learning ranks third, underscoring its importance in safeguarding personal data without direct data sharing. Additionally, support vector machines (SVM) for intrusion detection and AI applications in IoT security appear frequently, further highlighting the role of AI in safeguarding both traditional networks and emerging technologies like the Internet of Things.

Other areas like UAV system security and AI for mitigating DDoS attacks represent about 6%−7% of the focus, indicating moderate attention toward securing more specialized areas such as unmanned aerial vehicles and distributed denial-of-service mitigation. Topics such as blockchain for decentralized security, adversarial machine learning, and privacy-preserving data mining techniques occupy smaller percentages, around 4%−5%. This may suggest these fields are either in the early stages of adoption or considered more niche within the broader AI-cybersecurity landscape. Deep reinforcement learning and cryptography using ML also hold notable positions, contributing to the advancement of defensive mechanisms in critical infrastructure. The distribution showcases a wide array of AI applications in cybersecurity, with the potential for further exploration in less concentrated areas like ontologies in cybersecurity and AI in access and authorization, which accounted for lower percentages in the dataset.

### 3.2 Evolution of topics (RQ1)

**Figure 3:**
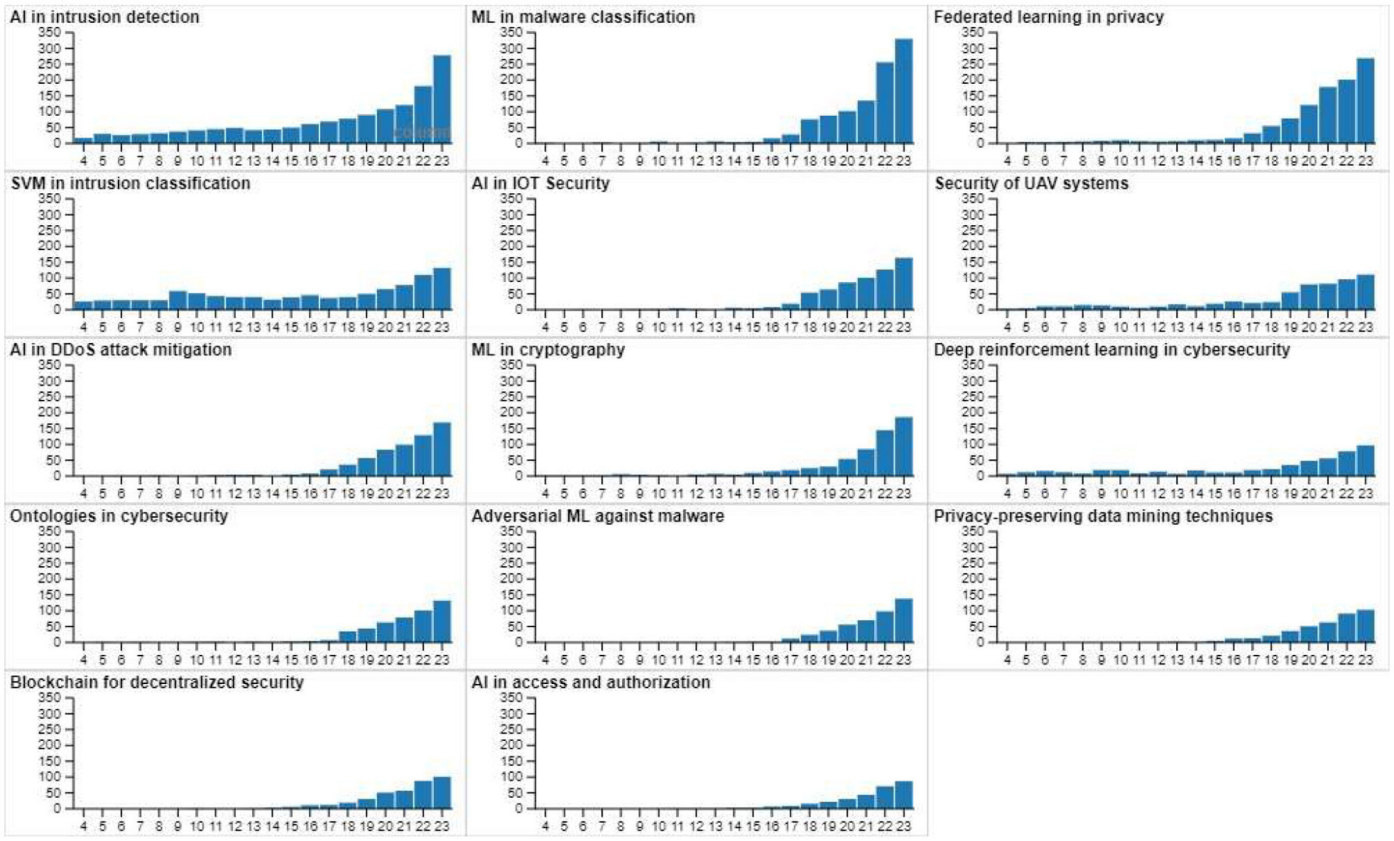
Evolution of major topics.

The visual representation in Figure 3 highlights the distribution of publications across various cybersecurity topics by year. The x-axis represents the years from 2004 to 2023. AI in intrusion detection dominates as the most widely researched area, with a substantial number of publications starting from the mid-2010s and accelerating in recent years. The focus on intrusion detection reflects the ongoing need to develop advanced techniques to identify and mitigate unauthorized access to systems, underscoring the significance of AI in addressing this pressing cybersecurity challenge. Machine learning (ML) in malware classification also shows a steady increase in publications, mirroring concerns over the rise of malicious software and its evolving complexity. The surge in research aligns with the growing need for more sophisticated methods to detect and classify different types of malware. These two areas—intrusion detection and malware classification—are clear priorities within the cybersecurity field, as evidenced by their consistently high number of publications. Federated learning in privacy exhibits a more gradual rise but has demonstrated significant attention over the past decade, especially as privacy concerns grow with the increase in distributed data systems. The role of SVM in intrusion detection and AI in IoT security shows a similar trajectory, indicating a balanced interest in applying AI to secure both traditional network systems and emerging technologies like the Internet of Things. Research on the security of UAV systems and AI in DDoS attack mitigation appears to have gained momentum post-2015, reflecting growing interest in specialized cybersecurity domains as these technologies become more integral to critical infrastructures. The exploration of deep reinforcement learning and ML in cryptography continues to grow, but their representation remains lower compared to core topics like intrusion detection. These areas are likely to gain further attention as AI-based solutions for cybersecurity become more advanced. Emerging fields like adversarial ML, ontologies in cybersecurity, and blockchain for decentralized security show a comparatively smaller number of publications, suggesting they are more niche or still developing in terms of research focus. Similarly, AI in access and authorization and privacy-preserving data mining techniques demonstrate increasing interest, albeit at a slower pace. Overall, the trend reveals a shift toward more diversified applications of AI in cybersecurity, with a clear emphasis on intrusion detection and malware classification, while newer areas like decentralized security and adversarial ML gradually gain traction.

### 3.3 Global distribution of topics (RQ2)

**Figure 4:**
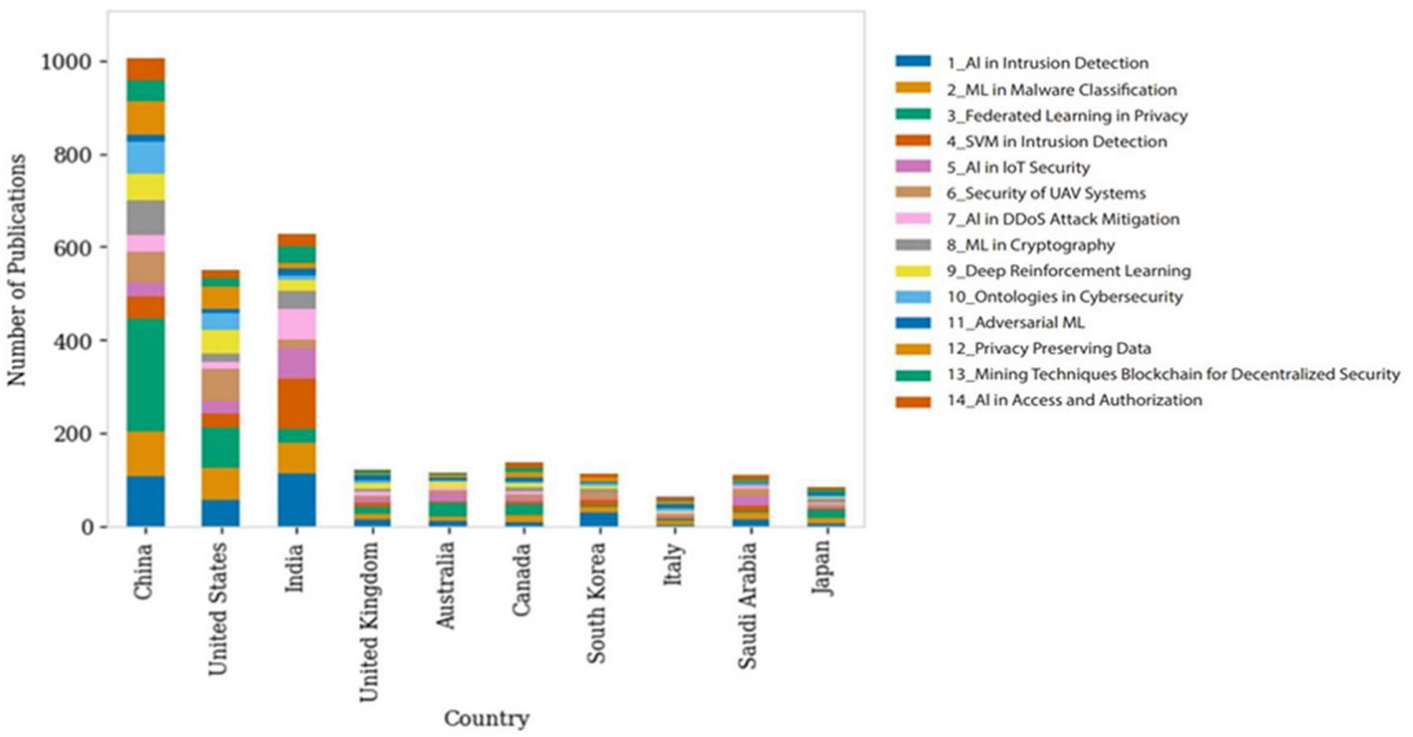
Global distribution of topics.

Figure 4 presents the global distribution of cybersecurity related publications, categorized by the affiliation of the corresponding author. The top 10 contributing countries organize the data and are further segmented by specific cybersecurity topics. It reflects the worldwide distribution of computer security analysis, revealing both the breadth of interest in the discipline and differing levels of attention given to specific subjects exhibited by different countries that might have been influenced by economic development, technological infrastructure, and policy preferences. The United States and the United Kingdom are two leaders from the Global North and have many publications on all topics, with much dominance in fields such as intrusion, malware, and system vulnerabilities. Their research spans a range of topics, from detailed technical aspects of cyber security, such as malware, SVM-based intrusion detection, and encryption, to broader issues, such as policy and authorization security. In fact, China is considered to belong to the Global South, but it is rapidly emerging as the world's leader in technology and has numerous publications on intrusion detection using DNNs and federated learning for privacy. This may be indicative of a strategic focus on developing advanced AI capabilities for cybersecurity. This figure is two to six times greater than that of other countries interested in AI, as per other findings (Han et al., 2021). The presence or absence of publications from Chinese institutions could show how far they have come regarding AI adoption for securing cyberspace. India, another major country from the Global South, has also made significant contributions to cybersecurity research across various topics while ensuring that it is well-balanced. There are also smaller contributions from countries such as Australia and Canada alongside other nations that belong to the Global North; however, these contributions do not compare to those made by China, the US or the UK concerning these topics. In contrast, some nations, such as South Korea, Italy, Saudi Arabia, and Japan, which are both part of the Global North and South, only present fewer publications, with some topics being given more attention than others, probably indicating that there may be certain specialized niches or growing interests in this field within such countries. Blockchain technologies lack productivity efficiency, and privacy innovations seem to receive less coverage compared with other subjects. In general, the chart shows not only how much output comes from each country regarding cybersecurity but also their areas of expertise.

### 3.4 Proximity of topics (RQ2)

An analysis of similarity was conducted to discern the proximity between the 14 topics in the context from which these data were derived (Kousis and Tjortjis, 2023). Topics such as intrusion detection by deep neural networks (DNNs) were found to be highly related to machine learning classifiers such as support vector machines (SVMs) for intrusion detection. This shows that machine learning techniques are central in present-day cybersecurity strategies for detecting intrusions. On the other hand, more specialized subjects, such as blockchain technology, which supports digital currencies such as Bitcoin and Ethereum, revealed less resemblance with other cybersecurity aspects, such as unmanned aerial vehicle (UAV) security or DDoS attacks. This suggests that within security domains, blockchain is regarded as a separate field with unique information protection criteria distinct from many others. Interestingly, privacy algorithms aimed at safeguarding individual identity appeared to be moderately similar to those involving authorization and access control. This might indicate that both have common concerns about securing user data and keeping information access well managed. In summary, the similarity matrix suggested a network where certain elements are closely tied through common approaches, such as advanced machine learning algorithms, while others remain more distinctive with special vulnerabilities. To effectively address the most pressing challenges in cybersecurity, understanding these connections is vital for guiding research and development investments.

### 3.5 Major topics of research (RQ3)

In the following sections, we discuss and summarize highly cited and relevant papers on different topics that have been identified under the BERTopic modeling of an entire dataset.

#### 3.5.1 AI in intrusion detection

There is a constant debate on the most effective method for intrusion detection in cybersecurity. According to Yin et al. (2017), recurrent neural networks (RNNs) should be employed in intrusion detection. These RNNs outperform both traditional machine learning techniques and other deep learning methods for binary and multiclass classification tasks. Ding and Zhai (2018) argued that deep learning, particularly convolutional neural networks (CNNs), is better than conventional machine learning approaches such as random forests and support vector machines. CNNs work well with large amounts of complex data on modern cyber traffic, and their empirical results support this fact. On the other hand, Kumar et al. (2022) offered a broader perspective in which they blended nature-inspired algorithms with deep learning techniques. The authors stress its efficacy in data processing and energy consumption, particularly in cloud environments, through their ant colony optimization (ACO), and deep neural network (DNN) models. All these studies provide strong arguments, but the bottom line is that deep learning methods, i.e., CNNs, RNNs, or DNNs, are more commonly perceived as potential future tools for effective intrusion detection within ever-changing cybersecurity world orders. While deep learning models like CNNs, RNNs, and DNNs are known for their accuracy, their resource-intensive nature remains a drawback. RNNs, despite handling sequential data well, suffer from training instability and require significant computational power, which may hinder real-time applications. CNNs, although powerful in data-heavy scenarios, face scalability issues when adapting to rapidly evolving intrusion patterns. The integration with nature-inspired algorithms, such as ACO with DNNs, promises efficiency but adds complexity, making the practicality of these hybrid models in dynamic and diverse environments questionable. As AI-driven approaches advance in cybersecurity, there is a growing need to explore technologies capable of processing vast amounts of data with unprecedented efficiency. Quantum computing offers promising solutions, particularly in enhancing AI's capabilities for complex threat detection and secure data handling.

#### 3.5.2 ML in malware classification

New and more sophisticated techniques are needed to detect the increasing complexity of malware. Byte-related deep learning, which challenges widely used API-based features, was proposed by Jung et al. (2018). They argued that their technique using byte information derived from images from malware outperforms the conventional CNN models with an astonishing 99% accuracy rate. Nevertheless, Snow et al. (2020) differ on this point and instead promote an end-to-end deep learning framework that incorporates several neural network architectures. In addition, they focused on how the architecture is capable of directly learning from input data, thereby enabling it to achieve state-of-the-art performance with improved training efficiency. Moreover, Gayathri and Vijaya (2021) suggested a CNN-based model for malware family classification. They also noted that there are limitations to traditional detection systems in regard to classifying new or polymorphic malware infections. According to them, over 95% accuracy can be realized in determining malware families by teaching a program based on pictures of executable files infected by malicious software, thus indicating that this approach is better than most present approaches for achieving this goal. All these studies provide well-reasoned explanations of the methodologies employed, revealing changes and complexities occurring within modern computer virology research. Although byte-level deep learning and CNNs offer high accuracy in malware classification, they often lack generalizability, particularly with novel or polymorphic malware. The byte-based approach is highly specialized, potentially limiting its adaptability across varying malware types. Furthermore, while end-to-end architectures enhance training efficiency, they may struggle with the ever-growing malware dataset sizes, raising concerns about the sustainability of current model performance without substantial computational investment. Real-time adaptability and efficient model updates remain challenging, particularly for models heavily reliant on static image features.

#### 3.5.3 Federated learning in privacy

Wei et al. (2020) dispute the vulnerability of deep learning systems to adversarial attacks and propose an attack-independent and self-repairing defense that transforms inputs strategically used for decision-making on adversarial instances. They asserted that this technique has greater efficacy in preventing attacks with fewer false positives than existing defenses. Fisichella et al. (2022) introduced a novel concept of partially federated learning by merging machine learning with federated learning for better privacy preservation. They claim that it is more adaptive to classical federated learning strategies because it enables clients to make flexible judgments about data sharing; such an approach enhances the performance earned over classical models. Nonetheless, Fontenla-Romero et al. (2023) argue against the belief that security is intrinsic in federated learning by pointing out its susceptibility to privacy leakages. They also suggested that the FL method can be improved using homomorphic encryption, which is claimed to be effective in training against attacks while preserving accuracy even on large datasets and multiple clients across networks. These studies collectively highlight the continuous struggle and creativity in securing AI systems from new threats while ensuring data privacy. Privacy leaks and adversarial attacks still threaten federated learning models, especially given the interdependencies of client data across networks. Techniques like homomorphic encryption attempt to mitigate this but introduce their computational demands, potentially impacting model accuracy and response time. Partially federated learning offers flexibility, but as data diversity increases, achieving uniform performance across clients without compromising privacy or efficiency proves to be a significant hurdle.

#### 3.5.4 SVM in intrusion classification

Thaseen and Kumar (2014) challenge traditional IDS approaches by proposing a novel integration of Principal Component Analysis (PCA) and Support Vector Machine (SVM), claiming superior accuracy and reduced resource consumption. Their method, they argue, outperforms other classification techniques, especially in identifying minority attacks. In contrast, Ahanger et al. (2021) emphasized the efficacy of machine learning methods such as random forest and SVM in IDS, which yielded a classification accuracy of over 99%. They advocate for feature selection to reduce computational complexity. Pardeshi and Patil (2023) highlighted deficiencies in IDS systems developed with outdated datasets. They propose training various machine learning models, including shallow learning algorithms, on updated datasets such as the NSL-KDD and CICIDS-2017 datasets to improve the accuracy, precision, recall, and F-measure. Each study presents a compelling case for their respective methodologies, underscoring the ongoing evolution and complexity in IDS research. Traditional methods, such as random forests and SVMs, can become computationally burdensome when updated with extensive feature sets, leading to performance trade-offs. Furthermore, reliance on outdated datasets like NSL-KDD risks overlooking recent attack vectors, emphasizing a need for models that can continually learn from real-time data to avoid obsolescence in a dynamic threat landscape.

#### 3.5.5 AI in IoT security

The rapid expansion of IoT devices has significantly increased vulnerability to cyberattacks, necessitating advanced security solutions. Ullah and Mahmoud (2021) addressed this issue by developing a novel anomaly based intrusion detection system (IDS) using CNNs for IoT networks. Their model, which is capable of multiclass classification, demonstrated high accuracy and efficiency using datasets such as BoT-IoT and IoT-23. In a subsequent study, Ullah and Mahmoud (2022) extended this approach by incorporating RNNs, including LSTM and GRU techniques, into their IDS. They proposed a hybrid model combining CNNs and RNNs, achieving notable success in both binary and multiclass classifications across various IoT datasets. Complementing these studies, Debnath et al. (2023) presented a comprehensive analysis of IoT security, proposing a seven-layer architecture to enhance IoT security. They categorize security threats at each layer and implement machine learning and deep learning algorithms, such as random forest and artificial neural networks, to mitigate these threats effectively. Together, these studies underscore the critical need for and effectiveness of advanced machine learning and deep learning techniques in securing increasingly complex IoT environments. CNNs may struggle to handle IoT network diversity, where lightweight and flexible models are needed for constrained devices. Additionally, while a seven-layer IoT security architecture is thorough, its complexity may limit its practical adoption, particularly in resource-constrained IoT devices where efficiency is paramount. The need for adaptable yet efficient models for secure IoT implementation remains an unresolved challenge in this field.

#### 3.5.6 Security of UAV systems

The use of drones in various sectors has raised serious concerns about cybersecurity. In their work, Dahiya and Garg (2020) focused on the threats posed by cybercriminals to UAVs with a focus on communication security. They identified crucial risks, such as Wi-Fi attacks and GPS spoofing, among others, thus stressing the importance of comprehensive prevention measures for UAV operations at different levels. In addition, Tao et al. (2021) proposed deep reinforcement learning for enhancing security in UAV networks. Such areas of concentration include the detection of hostile actions with respect to aerial computing networks pertaining to unmanned aerial vehicles, revealing how cutting-edge AI techniques could help fortify UAV applications. Additionally, Zhang Y. et al. (2023) presented a new lightweight authentication scheme using a physical unclonable function (PUF) that provides security for UAV links. This is due to its robustness against capture and impersonation, thereby providing a reliable method of ensuring the confidential sharing of data within UAVs without being compromised by adversaries who may seize control over them physically. The application of AI to UAV security is still nascent and faces several critical limitations. Techniques like DRL require extensive training and computational resources, which may not be feasible for lightweight UAVs. Furthermore, while PUFs provide a promising solution for authentication, their robustness against emerging threats, such as quantum attacks, is not well-established. Given the sensitivity of UAV operations, particularly in military or surveillance contexts, more rigorous testing and validation of these models are essential before real-world deployment.

#### 3.5.7 AI in DDoS attack mitigation

The increasing use of application-layer distributed denial of service (DDoS) attacks has been an area of concern in research. The ineffectiveness of traditional IP and TCP layer-based detection methods for addressing the complexity involved in application layer DDoS attacks was demonstrated in Yadav and Subramanian (2016). They propose that a more sophisticated deep learning architecture, particularly AutoEncoders, can be used to recognize intricate patterns in attacks, as they claim superiority over existing systems. Cil et al. (2021) emphasized the ability of DNNs, which are able to detect DDoS attacks with success rates as high as 99.99%, even when small samples are involved. Furthermore, Chen et al. (2021) argued that conventional intrusion detection systems are no longer applicable due to the growth in modern DDoS attacks and suggested CNNs as a possible means for their classification. They dispute the accuracy of other models, such as SVM and random forest, stating that CNN is better than them all. All these studies contend that there should be a strong alteration toward advanced deep learning practices for effectively addressing the evolving nature of DDoS attacks. While advanced deep learning architectures like AutoEncoders and CNNs show promise for DDoS detection, they may struggle with real-time responsiveness, especially during large-scale, high-speed attacks. Deep learning's high accuracy comes at the cost of significant computational overhead, which raises concerns about practical deployment in resource-constrained environments. Moreover, DNNs, while effective, are highly sensitive to data variations, which could reduce detection accuracy as attack methods evolve. These limitations point to the need for lightweight yet adaptable solutions in DDoS mitigation.

#### 3.5.8 ML in cryptography

The use of artificial neural networks in cryptography is occurring at a rapid pace. Santhanalakshmi et al. (2015) argued that neural cryptographic effectiveness can be achieved using tree parity machine neural networks for key generation. They stress the importance of mutual learning and neural synchronization for creating cryptographic key exchange protocols supported by genetic algorithms for faster synchronization. Fang et al. (2022) questioned conventional image encryption techniques by suggesting a new combination of hyperchaotic systems and generative adversarial networks (GANs) for digital image encryption. Their technique allegedly provides greater security, stronger robustness, and more space for keys. Dhakne et al. (2022) further support this view by pointing out that neural cryptography enables symmetric encryption and prevents brute force attack threats. They envisioned the next level of cryptography using neural networks, which would give rise to highly secure and attackproof cipher systems. These studies collectively indicate an increase in reliance on neural networks to offer leading-edge, secured cryptographic solutions. Machine learning in cryptography, especially neural networks, is innovative but remains vulnerable to emerging threats like quantum computing. While neural cryptographic methods using Tree Parity Machines and GAN-based encryption offer advancements, they are untested in high-stakes environments. Moreover, these methods often require extensive parameter tuning, which limits their accessibility and adaptability. This reveals a key weakness in the current ML-based cryptography models: they provide theoretical security benefits but lack practical, widespread adoption due to their complexity and computational demands.

#### 3.5.9 Deep reinforcement learning in cybersecurity

DRL, which is being employed in cybersecurity, has revolutionized the way modern systems shield themselves from cyberattacks. A new DRL-IDS employing Markov decision processes and stochastic game theory was suggested by Benaddi et al. (2022). Their method shows a higher detection rate and accuracy than traditional methods, which causes false alarms to occur more effectively. Oh et al. (2023) show even more how DRL can be utilized for cybersecurity by the use of a DRL framework within adversarial cyber-attack simulations. Their agent-based model adapts to changes in the network environment, thereby surpassing existing techniques in learning optimal ways of attacking. Nguyen and Reddi (2021) presented a comprehensive survey on applications of DRL in cybersecurity that included its effectiveness in cyber-physical systems, autonomous intrusion detection, and multiagent game theory simulations. Together, these studies emphasize the ability of DRL to handle the complexity and dynamics of threats, providing an encouraging direction for future solutions to cybersecurity problems. DRL has a significant potential to revolutionize cybersecurity by offering adaptable, self-optimizing models. However, DRL models are notoriously data-hungry and require extensive training cycles, which can be prohibitive in rapidly changing environments. The complexity of DRL's Markov decision processes may also lead to overfitting, making the models less effective when faced with novel attack patterns. Additionally, DRL's computational requirements limit its feasibility in resource-limited settings, indicating a need for more efficient DRL approaches to bridge the gap between accuracy and practicality.

#### 3.5.10 Ontologies in cybersecurity

A number of studies have been conducted on the effectiveness of ontology-based knowledge systems in addressing complex cyber-attacks such as advanced persistent threats (APT) or IoT security vulnerabilities. Kim et al. (2019) recommend that a three-part ontology structure be employed to connect APTs, general security, and domain-specific knowledge since these structures are often multifaceted. This approach will help in tailoring the specific security requirements for different attack scenarios. However, Zhang et al. (2021) addressed IoT security by introducing the IoT Security Threat Ontology (IoTSTO) for managing heterogeneity and complexity in IoT environments. According to these authors, its framework offers a holistic approach to analyzing IoT security. Ren et al. (2022) provide an alternative perspective by emphasizing open-source cyber threat intelligence together with knowledge graphs for the identification and mitigation of APTs. They suggested that CSKG4APT goes beyond passive defense by actively integrating and updating threat intelligence. Such studies make strong cases for each ontology-driven approach toward developing cybersecurity strategies. Ontology-driven approaches bring a structured method to cybersecurity (Kannimoola et al., 2021). As threat landscapes evolve, ontologies must be updated regularly, which can be resource-intensive and slow to adapt. Frameworks like IoTSTO are limited by their reliance on comprehensive, updated threat intelligence, which may not always be available. Furthermore, these frameworks often struggle with heterogeneity, limiting their scalability across diverse IoT and APT scenarios. The effectiveness of ontology-driven solutions, while promising, is constrained by their high maintenance requirements.

#### 3.5.11 Adversarial ML against malware

In regard to the development of well-functioning malware detectors, there is much contention, with most recent research pointing out the challenges and advancements in this area. Reinforcement learning (RL) and GAN by Phan et al. (2022) are used in developing adversarial malware samples that bypass ML detectors and VirusTotal. As such, their approach trains an RL agent to mutate malware, thus improving the resilience of malware detectors against metamorphic malware. Arif et al. (2023) suggested IF-MalEvade, an evasion framework that combines a GAN with DRL to create functional malware samples with differences such as head section manipulation. This approach effectively circumvents black box ML detectors while preserving their function. A novel query-free approach was proposed by Gibert et al. (2023), which uses a GAN-based framework to generate adversarial malware examples that mimic benign executables in feature space. Hence, this technique reduces the detection efficiency of up-to-date antimalware engines and state-of-the-art malware detectors without first querying them as viewed from experiments on various systems. These investigations illustrate the changing face of cyber security threats, calling for more resilient systems underpinned by machine learning techniques for spotting malicious code. GANs and RL models create sophisticated adversarial samples that may evade detection, but these models themselves require regular updates to stay effective. Additionally, evasion frameworks like IF-MalEvade demand significant resources and time to generate adversarial samples, questioning their practical applicability in real-time detection. As these systems grow more advanced, their maintenance, complexity, and dependency on large datasets could impede scalability and real-world feasibility.

#### 3.5.12 Privacy-preserving data mining techniques

Privacy-preserving data mining (PPDM) techniques aim to solve the problem of extracting knowledge from data while protecting sensitive information. Bertino et al. (2005) discussed different PPDM algorithms for hiding sensitive item sets and avoiding the possibility of predicting sensitive data via classification approaches. For this purpose, they advocate for a holistic assessment framework that can be used to appraise these algorithms' effectiveness in terms of privacy protection as well as maintaining data integrity. Gadepaka and Surampudi (2015) contributed to this area by proposing privacy-preserving collaborative clustering using self-organizing maps (SOMs) for distributed data where multiple parties can work together without revealing their data directly. In their further study, Gadepaka and Surampudi (2021) expanded on PPDM by proposing new algorithms concerned with horizontal and vertical partitioning of data using cryptology and perturbation, respectively. They demonstrate how far secure algorithms and models have been in developing the PPDM field with respect to individual privacy during various mining activities on different kinds of datasets. PPDM techniques like collaborative clustering with SOMs offer secure data sharing but suffer from trade-offs between data utility and privacy. Cryptographic partitioning methods are computationally intensive, and as datasets scale, maintaining efficiency becomes increasingly challenging. These PPDM models may also fall short in protecting against inferential attacks, where adversaries deduce sensitive information indirectly. Although effective in principle, these techniques struggle to balance privacy with performance, highlighting an ongoing challenge in ensuring robust, privacy-preserving data mining.

#### 3.5.13 Blockchain for decentralized security

Gul et al. (2020) detail how the use of blockchain in healthcare could be employed to automate tasks and to provide better security and performance. In particular, the authors discuss how reinforcement learning can be used to train agents with regard to the efficient management of blockchain in healthcare. Haddad et al. (2021) proposed a combined solution for machine learning and blockchain applications in healthcare data administration involving convolutional neural networks (CNNs), which carry out intelligent data extraction as well as diagnosis, and blockchain technology, which ensures the secure sharing of such information. Aggarwal et al. (2022) DeMed is a decentralized framework for medical image analysis based on blockchain. It finds its strength by combining self-supervised learning on local devices with smart contracts on the ledger, thereby validating its effectiveness in medical image classification tasks. By this integration, these studies strive to achieve better health research, health care provision and privacy of information through the combination of both techniques represented by machine learning algorithms and blockchain technologies. While blockchain provides security and transparency in healthcare, it faces scalability and data latency issues that hinder real-time processing. Integration with machine learning, as seen in the DeMed framework, adds complexity and requires high computational resources, which may limit its deployment in under-resourced settings. Additionally, as blockchain networks grow, maintaining decentralized consensus becomes harder, and transaction costs increase, posing challenges for sustainable long-term adoption. Thus, blockchain's role in healthcare security, while promising, must address scalability, speed, and cost limitations to be widely practical.

#### 3.5.14 AI in access and authorization

Advanced frameworks for enforcing and analyzing access control policies in different computing environments have been discussed (Rao et al., 2009; He et al., 2010; Liu et al., 2019). The semantic web framework with a meta-control model introduced by Rao et al. (2009) is used to enforce complex policies. For dynamic service discovery and access, this framework uses policy-enhancing agents and semantic web reasoning, which expands the XACML architecture to cover diverse policy scenarios. A context and role-based access control policy model for web services was suggested by He et al. (2010) that uses description logic to formalize policies while employing a knowledge base for policy reasoning; the Racer reasoning system validated this model. A constraint-based framework called CACPSAF that provides a security analysis of access control policies was presented in Liu et al. (2019). This implies that the analysis of security principles based on policy security is transformed into solving for satisfiability variables while maintaining incremental checking of access controls through fine-grained scrutiny processes to guarantee their accuracy. The articles have pointed out the necessity of advanced mechanisms for controlling and scrutinizing compliance in various computing environments that facilitate secure yet efficient access control systems. Advanced AI frameworks for access control, like semantic web-based policies, offer control but are challenging to implement at scale. The reliance on complex meta-control models and context-based rules increases their maintenance burden, making them susceptible to inconsistencies. Additionally, access control models based on knowledge bases can become outdated if not continually refined, which risks creating security gaps. Ensuring the accuracy and responsiveness of these access control systems in dynamic environments remains a critical challenge for AI-based authorization frameworks.

## 4 Implications and future research directions (RQ4)

The application of AI in cybersecurity has grown significantly, with diverse approaches being explored to combat emerging cyber threats. As such, it is vital to understand the implications of these findings for both policymakers and practitioners, who play key roles in shaping the future of cybersecurity.

### 4.1 Implications for policymakers

Policymakers must recognize the increasing role of AI in intrusion detection as a cornerstone of cybersecurity strategies. Given the demonstrated efficacy of deep learning techniques such as RNNs, CNNs, and hybrid models, it is crucial to allocate funding for research and development in trustworthy AI (Gardner et al., 2022). Moreover, policies should encourage the adoption of these advanced techniques in critical infrastructure protection, mandating periodic evaluations and updates to ensure that systems remain resilient against evolving cyber threats. For malware classification, policymakers need to push for regulations that standardize the use of AI-driven methods across industries to enhance detection capabilities (Jeon, 2024). Given the increasing complexity of malware, it is important to promote cross-border collaborations that facilitate the sharing of data on emerging threats, allowing AI models to evolve and detect sophisticated forms of malware. This can also be supported by creating guidelines for ethical AI use in cybersecurity to protect personal and organizational data during these processes. Federated learning in privacy offers a critical opportunity for policymakers to balance innovation with privacy. Policymakers should promote frameworks that ensure the security of federated learning environments, especially in sectors such as healthcare and finance, where privacy is paramount (Kaissis et al., 2020). Policies need to incorporate encryption standards, like homomorphic encryption, into legislation, ensuring robust privacy protections across distributed networks without stifling innovation (Dizon and Upson, 2021). Policymakers should invest in building open-access datasets and support research initiatives that refine current learning algorithms. Additionally, national cybersecurity strategies should emphasize updating security models to reflect the latest datasets and threat landscapes, ensuring adaptive and real-time protections.

### 4.2 Implications for practitioners

For practitioners, the application of AI in intrusion detection signals the need for integrating deep learning systems into existing security infrastructures. To maximize effectiveness, professionals should focus on optimizing AI algorithms for specific use cases, ensuring that these systems can handle large and complex data streams (Khamis, 2024). Regular retraining of models is essential, as evolving cyber threats—including adversarial attacks—can compromise detection accuracy if models are outdated. In malware classification, cybersecurity practitioners must adopt new techniques, such as byte-related deep learning models, to stay ahead of increasingly sophisticated malware threats. By leveraging CNN-based models and alternative methods like image analysis of malware, organizations can significantly improve malware detection and classification. Additionally, models should be hardened against adversarial inputs designed to bypass detection, particularly in the face of polymorphic malware (Catalano et al., 2022). For those working with federated learning in privacy, a major takeaway is the need to address vulnerabilities in distributed learning models. Homomorphic encryption and attack-independent defenses, such as differential privacy, are crucial for securing federated learning applications. Additionally, fostering collaboration between organizations can enhance model robustness against privacy leakages and adversarial manipulation, particularly when handling sensitive data (Wei and Liu, 2024). For those working with SVM in intrusion classification, integrating PCA and feature selection not only reduces resource consumption but also improves accuracy. Staying updated with the latest datasets and adversarial training practices can significantly enhance the robustness of SVM-based models, especially for detecting minority and novel attacks designed to evade traditional classifiers.

### 4.3 Future research directions

Building on the diverse research thematic areas explored in the earlier sections, we suggest several key avenues for future research. As cybersecurity faces increasing challenges, traditional computing methods are reaching their scalability limits, especially when confronting advanced, multifaceted cyber threats. Quantum computing, with its ability to process complex computations in parallel, is anticipated to offer transformative solutions in cybersecurity. By integrating quantum capabilities, particularly in encryption resilience and high-dimensional threat analysis, quantum-enhanced AI can address the intricate demands of protecting critical infrastructure in ways classical systems cannot achieve.

1. **Quantum Machine Learning (QML) in cybersecurity:** The integration of QML into cybersecurity, particularly for critical infrastructures, is a major leap forward against advanced cyber threats. QML's rapid processing capabilities should be exploited by having domain-specific quantum security protocols that may enable faster vulnerability detection and dynamic defense strategies (Rajawat et al., 2023). It is important that vulnerabilities in quantum systems are investigated to design robust cyber defense mechanisms that can fortify cybersecurity frameworks during the era of quantum computing.
2. **Resilience against quantum threats:** Resilient QML could be used as a basis for designing quantum networks that withstand adversarial attacks (West et al., 2023). Optimizing these networks for accuracy across different data types and reevaluating cryptographic methods such as quantum-resistant encryption and enhanced quantum key distribution (QKD) are vital areas of investigation for addressing adversarial weaknesses and scalability issues with quantum processors.
3. **Quantum AI (QAI) in cybersecurity education:** Incorporating quantum artificial intelligence (QAI) into cybersecurity education, especially within software engineering programs (Baldassarre et al., 2023), is an important endeavor to keep pace with developments in the field of quantum advancements. This strategy also targets preparing the cyber workforce for the age of quantum by ensuring that security measures keep up with both the increasing sophistication level of malicious programs and the increase in the number of threats originating from technology based on this principle.
4. **Explainable AI (XAI) in cyber threat analysis:** Advancing XAI for cybersecurity involves refining algorithms and evaluation metrics to improve threat identification while minimizing false positives (Rjoub et al., 2023). Going forward, efforts should be directed toward striking a balance between privacy and explainability within XAI systems through integrated approaches combining various attributes to enrich XAI explanations within complex cyber threat landscapes.
5. **Neuro-symbolic AI for enhanced cybersecurity:** Enhancing real-time threat detection for defending against advanced adversarial techniques is a hot area of research. Piplai et al. (2023) demonstrated promising research in this direction by combining the pattern recognition of neural networks with symbolic reasoning through neurosymbolic AI.
6. **Advancements in AI-Driven malware detection:** As cyber threats mutate, so should the AI tools that combat them. Studies should emphasize advancements in malware detection models using adversarial training and the development of smarter traffic analysis frameworks that eschew conventional manual feature extraction methods (Lucas et al., 2023; Qu et al., 2023), with the aim of improving the ability of these methods to adapt and be more accurate in handling attacks.
7. **Deep learning for cyber-physical systems security:** Deep learning (DL) plays a vital role in the security of cyber-physical systems (CPSs), ensuring network intrusion detection. Investigations of advanced DL architectures and methodologies such as federated learning for dataset limitations and improved threat detection capabilities are proposed to develop extendable and independent safety models for complex CPS ecosystems, next-generation networks, and IoT infrastructures. One potentially applicable field of IDS study is deep reinforcement learning due to its versatility and agility. For instance, deep-RL has been used against various adversarial attacks, such as the fast gradient sign method (FGSM) and the basic iterative method (BIM) (Zhang et al., 2021).
8. **Counteracting deepfakes:** Developing sophisticated countermeasures against deepfakes is an area to be researched. Leveraging transfer learning, data augmentation, explainable AI (XAI), or a combination thereof can ensure that model transparency is enhanced, thereby enabling robust deepfake detection. Knowledge fusion with advanced anomaly detection techniques combined with decision-making strategies is a dynamic defense against threats that endanger digital content integrity.
9. **Blockchain-AI fusion for IoT security:** Combining AI's predictive analytics with Blockchain's unchanging ledger will address cyber threats that are growing in IoT networks (Alharbi et al., 2022). Further research emphasizing privacy, trust, etc., should advocate for dynamic decentralized security systems that neutralize preemptive threats in IoT systems, highlighting privacy, trust, interoperability and sustainability in IoT systems, as well as AI-driven automated response mechanisms that enhance the resilience of the IoT.
10. **Enhancing privacy in federated Learning:** Building upon the differential privacy (DP) framework for federated learning (Zhang L. et al., 2023), which leverages edge intelligence, is a promising direction for enhancing privacy protection in distributed FL environments. Exploring the Federated Deep Relationship Prediction (FDRP) framework, especially in vertical FL contexts (Liu et al., 2024), to mitigate inference attack risks while maintaining high prediction accuracy and convergence rates has several unexplored challenges.
11. **AI powered metaverse cybersecurity:** As the Metaverse expands with VR and AR technologies, identifying and mitigating cybersecurity vulnerabilities has become a critical area for research (Chow et al., 2022). Therefore, robust countermeasures should be developed to protect against emerging threats to ensure a secure and seamless virtual experience that takes advantage of AI, such as content automation or user interaction, within these immersive landscapes.
12. **Domain specific AI in cybersecurity:** To strengthen defense mechanisms against sector-specific threats, it is suggested that AI solutions be tailored for sectors such as healthcare, finance, energy, and government. Regarding this issue, investigating the use of AI in cybersecurity within these domains suggests the need for adjusting to changes in regulations or countering the sophistication of AI-powered malware to guarantee the resilience of critical infrastructures and the integrity of national security information.
13. **Integrating humanized AI insights in cybersecurity:** Designing secure and user-friendly systems should involve suggestions from psychology, sociology or law. This provides a complete perspective on how effective AI solutions for cybersecurity are based on a comprehensive understanding of the human factors that affect cyber interactions. Measures must be not only technologically advanced but also synchronized with human behavior, social norms, and legal standards.
14. **Developing global standards:** Design and establish norms for AI applications in cybersecurity that are globally relevant. These standards should address various aspects, such as data processing, model training, accuracy of threat detection, and response protocols. Furthermore, this could aid in developing precise, quantifiable metrics to assess the accuracy of threat detection, the speed of response, the false positive rate, and the resilience of AI systems. The goal would be to create a universal framework that ensures the safe, ethical, and effective development and deployment of AI systems across different regions and sectors.
15. **Enhancing sustainable development goals through AI integration:** Focusing on the intersection of AI in cybersecurity and Sustainable Development Goals (SDGs) is important, particularly for SDG 9 (Industry, Innovation, and Infrastructure) and SDG 16 (Peace, Justice, and Strong Institutions), by safeguarding information infrastructure and promoting safe, secure societies (Achuthan et al., 2023; GSDR, 2023). This connection presents a vital area for future research, exploring how AI-driven cybersecurity strategies can contribute to achieving these SDGs while addressing the challenges of digital security and privacy.

## 5 Contributions and addressed gaps

### 5.1 Summary of key findings (RQ1)

The analysis for RQ1 underscores the significant application of AI in cybersecurity, particularly in intrusion detection and malware classification, reflecting the urgency to combat sophisticated threats. Intrusion detection dominates the research landscape with an accelerating number of publications from the mid-2010s, highlighting the ongoing development of advanced techniques to mitigate unauthorized access. Similarly, machine learning in malware classification exhibits steady growth in response to the increasing complexity of malware. Federated learning's role in privacy and AI's expansion into IoT security highlight the technology's versatility across cybersecurity domains. Emerging areas like blockchain and adversarial machine learning, though less represented, suggest potential growth sectors. Federated learning is gaining traction in privacy applications, demonstrating its importance in collaborative environments where privacy preservation is critical. The study also shows sustained interest in applying AI to secure both traditional networks and emerging technologies like IoT. Specialized areas such as UAV systems security and AI in DDoS attack mitigation have seen increased attention post-2015, indicating the growing relevance of AI in securing critical infrastructures. Although emerging fields like blockchain, adversarial machine learning, and ontologies in cybersecurity are less represented, they show potential as niche areas for future research. This comprehensive overview not only maps the current distribution and evolution of AI applications in cybersecurity but also identifies areas with growing interest, indicating a shift toward diversified AI applications and providing insights into future research trajectories.

### 5.2 Summary of key findings (RQ2)

Upon exploring RQ2, it reveals significant geographic and thematic variations in AI-driven cybersecurity research, with the US and UK leading in comprehensive topics such as intrusion detection and malware, while China excels in deep neural networks and federated learning, indicating a robust strategic focus on AI. Countries like India show balanced contributions, whereas others like Australia and Japan exhibit niche focuses. Thematic relationships within the field highlight machine learning's central role in intrusion detection, contrasting with blockchain's distinct niche. This synthesis not only provides a detailed mapping of global research outputs and thematic connections but also fills existing literature gaps by identifying how different regions contribute to and prioritize various AI cybersecurity topics. By delineating these patterns, the work advances the understanding of global and thematic trends in AI applications within cybersecurity, informing future research directions and policy formulations in the field.

### 5.3 Summary of key findings (RQ3)

Findings from RQ3 offers a focused analysis of fourteen major AI-driven cybersecurity topics, illustrating both the advancements and limitations within each area over the past two decades. In intrusion detection, AI techniques such as RNNs and CNNs have been paramount in enhancing detection accuracy but struggle with high computational demands and adaptation to new threats. Machine learning's role in malware classification has evolved with high-precision methods like byte-level deep learning, yet these face challenges in detecting polymorphic malware. Federated learning has progressed in securing privacy in distributed networks but is vulnerable to adversarial attacks and complex to implement effectively. In IoT security, the integration of CNNs and RNNs has fortified defenses but encounters issues with the diverse nature of IoT devices. UAV system security has benefited from deep reinforcement learning for threat detection; however, the computational intensity of these methods limits their practicality. DDoS attack mitigation has seen improvements with the use of DNNs and AutoEncoders, which excel in pattern recognition but may not respond swiftly enough in real-time attacks. Cryptography has advanced with AI through neural networks for secure key generation but remains susceptible to quantum decryption methods. Deep reinforcement learning has shown promise in creating adaptable cybersecurity defenses but requires substantial training data and processing power. Blockchain applications have been explored for decentralized security, particularly in healthcare, yet they face scalability and latency issues.

### 5.4 Summary of key findings (RQ4)

While addressing RQ4, exploration of emerging challenges and underexplored areas in AI-driven cybersecurity identifies several critical avenues requiring advanced methodologies and interdisciplinary approaches to foster scalable, resilient, and ethical solutions. The pressing need for quantum-enhanced AI methodologies to tackle advanced cyber threats highlights an area ripe for significant innovation, especially in quantum machine learning (QML) which promises to revolutionize cybersecurity practices with its superior processing capabilities. Similarly, the integration of neuro-symbolic AI and blockchain-AI fusion points toward the necessity for robust, dynamic systems that can adapt to evolving threats in real-time, particularly in complex networks like IoT. This study also underscores the potential of explainable AI (XAI) and AI-driven malware detection improvements to enhance transparency and adaptability in cybersecurity mechanisms. By proposing advanced AI applications such as federated learning that incorporate differential privacy and real-time adaptability in deep learning for cyber-physical systems security, this research outlines critical developmental pathways. Furthermore, addressing cybersecurity in the burgeoning context of the Metaverse and tailored AI solutions for sector-specific threats illustrates the broadening scope of AI applications that need to be explored. This analysis addresses the specified research question by clearly mapping out the landscape of emerging challenges and the potential of interdisciplinary approaches in AI cybersecurity. It advances the field by identifying underexplored areas such as quantum AI, neuro-symbolic AI, and AI in Metaverse security, proposing novel research directions that fill existing gaps in the literature. By highlighting these areas, the work not only guides future research trajectories but also informs policymakers and practitioners about the strategic priorities necessary for advancing AI-driven cybersecurity solutions.

## 6 Conclusions

As cyber threats become increasingly sophisticated, the role of AI in enhancing the efficiency and effectiveness of cybersecurity measures has shown significant promise, albeit shadowed by the apprehensions of its exploitation. The corpus of literature dedicated to this intersection has witnessed a meteoric rise, underscoring an imperative demand for a comprehensive synthesis. Through systematic and methodical literature analysis, a wide range of studies that addressed the development and application of AI in cybersecurity were included. BERTopic modeling and analysis of this dataset yielded 14 thematic areas, including how AI is utilized in intrusion detection, malware classification, privacy-preserving applications, IoT security, UAV systems, DDoS attacks, adversarial scenarios, and decentralized schemes, among others. Considering these insights, this study proposed several key areas for future research. These include exploring the integration of quantum machine learning in cybersecurity, particularly for critical infrastructure protection, and the development of domain-specific quantum security protocols. The need for advancements in explainable AI for threat analysis, the exploration of neuro symbolic AI for enhanced cybersecurity, and the development of sophisticated countermeasures against evolving threats such as deep fakes are highlighted as crucial avenues. Additionally, the study underscores the importance of tailoring AI solutions to sector-specific challenges and integrating human-centric insights into cybersecurity solutions, emphasizing the need for an integrated approach that incorporates psychology, sociology, and law.

Generative AI's ability to create adaptive, predictive models offers unparalleled opportunities for preempting cyber threats and automating defense mechanisms. However, it also presents challenges, particularly in the creation and detection of sophisticated cyber-attacks, such as deep fakes. The findings and insights from this extensive analysis have profound implications for policymakers, practitioners, and the broader cybersecurity community. For policymakers, understanding the evolving role of AI in cybersecurity is crucial for shaping regulations and standards that foster innovation while ensuring digital security and privacy. Practitioners can leverage these insights to adopt cutting-edge AI technologies and methodologies, enhancing their cybersecurity frameworks and response strategies. Our comprehensive investigation acknowledges certain limitations. The utilization of thematic clustering alongside BERTopic modeling, though effective in providing a structured analysis, may not encapsulate the intricate nuances of the cybersecurity domain completely, possibly resulting in a simplified portrayal of certain dimensions. Furthermore, the interpretive process inherent in delineating themes and topics, despite the sophistication of the modeling techniques employed, remains susceptible to subjective biases or potential misinterpretations. This underscores the importance of rigorous scrutiny and corroboration by domain experts to ensure the accuracy of the derived insights.

Our study has certain limitations. Within the PRISMA framework, potential biases may arise from selecting literature from a single database. With respect to BERT topic modeling, we recognize its ability to manage large datasets and identify key themes. By generating coherent topic representations based on the semantic similarity of words and phrases, topic modeling helps reduce human biases in topic classification, thus enhancing the objectivity and reliability of the findings (Kimura, 2024). However, the effectiveness of these algorithms can be influenced by the quality of the input data and the assumptions inherent in their design.

## Data Availability

The raw data supporting the conclusions of this article will be made available by the authors, without undue reservation.
